# Roles and Regulation of Quorum Sensing of Acidophiles in Bioleaching: A Review

**DOI:** 10.3390/microorganisms12030422

**Published:** 2024-02-20

**Authors:** Wang Luo, Yiran Li, Shiqi Chen, Yili Liang, Xueduan Liu

**Affiliations:** 1 School of Minerals Processing and Bioengineering, Central South University, Changsha 410083, China; luowang@csu.edu.cn (W.L.); yiranli@csu.edu.cn (Y.L.);; 2 Key Laboratory of Biometallurgy, Ministry of Education, Changsha 410083, China

**Keywords:** quorum sensing, N-acyl homoserine lactones, biofilm formation, bioleaching

## Abstract

Bioleaching has gained significant attention as a cost-effective and environmentally friendly approach for extracting metals from low-grade ores and industrial byproducts. The application of acidophiles in bioleaching has been extensively studied. Among the various mechanisms leaching microorganisms utilize, quorum sensing (QS) is pivotal in regulating their life activities in response to population density. QS has been confirmed to regulate bioleaching, including cell morphology, community structure, biofilm formation, and cell metabolism. Potential applications of QS have also been proposed, such as increasing mineral leaching rates by adding signaling molecules. This review is helpful for comprehensively understanding the role of QS in bioleaching and promoting the practical application of QS-based strategies in bioleaching process optimization.

## 1. Introduction

Bioleaching is a process that involves the use of microorganisms to extract valuable elements from ores [1]. This technology offers a range of benefits such as low cost, short processing time, environmental friendliness, and minimal pollution [2]. As a result, bioleaching has become a leading technology in the field of mineral processing around the world [3]. Many acidophilic microorganisms thrive in acid mine drainage, exhibiting remarkable adaptability, and can be applied to bioleaching and mitigating heavy metal contamination [1,4,5,6].

Microorganisms dissolve metals by non-contact or contact mechanisms [7,8]. Acidophilic microorganisms use inorganic sulfur compounds to produce sulfate while converting Fe^2+^ and Fe^3+^, playing important roles in global sulfur and iron cycling (Table 1) [9,10]. Over the last decade, there has been a gradual increase in the discussion surrounding leaching biofilms [11,12,13,14,15]. The attachment of cells to solid surfaces is an essential step in initiating bioleaching, as it forms the biofilm and is critical to the mineralization process [16]. Bacterial cells attached to surfaces produce extracellular polymers (EPS) associated with biofilm formation [17]. This process is thought to be mediated by quorum sensing (QS) [18].

QS is an important mechanism by which microorganisms regulate their life activities based on population density [26]. When the number of microorganisms in an area increases enough, they exhibit “social” behavior. These microorganisms use autoinducers (AIs) secreted into their environment to determine their population density. As the density of the bacterial population increases, the concentration of AIs also increases in the surrounding environment [27]. AIs are transported across the cell membrane, where they bind to corresponding receptors. This binding initiates a cascade of reactions regulating physiological and biochemical processes, such as root nodulation, bioluminescence, protein secretion, motility, virulence factor production, plasmid transfer, and biofilm formation [28,29,30].

According to different AIs, the QS pathways are divided into seven types. AI oligopeptides are found in Gram-positive bacteria [31]. The remaining six signaling pathways are distributed in Gram-negative bacteria: AHL (AI-1), AI-2, and CAI-1, first discovered in *Vibrio* [32,33], PQS signaling in *Pseudomonas aeruginosa* [34], AI-3 signaling in *Escherichia coli* O157:H7 [35], and DSF in *Xanthomonas campestris* [36]. QS commonly found in biofilm formation has also been observed in bioleaching systems [37]. In the early 21st century, *Acidithiobacillus ferrooxidans* was reported to have a functional N-acyl homoserine lactones (AHL) QS system by Farah et al. [38]. Rivas et al. discovered that *A. ferrooxidans* has another QS system, an atypical one consisting of *glyQ*, *glyS*, *gph*, and *act* genes [39]. Bellenberg et al. demonstrated an interspecies QS signaling mechanism for functional diffusion signaling factor (DSF) production by *Leptospirillum ferriphilum* and *Leptospirillum ferrooxidans* [40]. In addition to AHLs, acidophiles have enzyme systems encoding second messenger cyclic di-guanosine (c-di-GMP) that assist in regulating the QS system [41]. Huang et al. revealed the presence of AHL, autoinducer-3, DSF, and AHL-degrading enzymes by annotating the genome of 83 species of acidophiles [42]. Bioinformatics prediction and laboratory experiments have identified the QS system in acidophiles.

However, the role and function of QS in bioleaching systems have not been systematically summarized and discussed. Therefore, we searched the literature from the Web of Science database by using the keywords “bioleaching”, “acidophiles”, and “quorum sensing”, screened more than 120 documents that fit the topic, and reviewed the theoretical and practical understanding of QS in bioleaching. This paper provides a brief overview of the QS mechanisms in acidophiles, describing in detail the interconnections between the different mechanisms. Then, its role in cell morphology, community structure, biofilm formation, and microbial metabolism is summarized, emphasizing the exogenous regulation of QS on various bioleaching processes in acidophiles. The challenges and future development directions of QS-based control strategies are discussed. This information shows the great potential of QS in bioleaching regulation and contributes to the further development of QS-based control technologies.

## 2. QS and Second Messenger in Acidophiles

At present, the main QS systems studied in acidophiles are AHL and DSF systems, and c-di-GMP systems related to QS systems are also partially studied (Table 2). The same bacterium can contain multiple QS systems at the same time, and there are cascade, parallel, and competitive relationships between different systems. Different bacteria can communicate by generating, binding, and degrading the same AIs to adapt to extreme environments and improve competitiveness.

### 2.1. N-Acyl Homoserine Lactones System

The most extensively studied QS system in acidophiles is the AHL type. This system consists of at least four elements: AHLs, AHL synthase proteins, transcriptional regulators belonging to the family of R proteins, and cis-activated palindromic DNA sequences that serve as targets for R-AHL binary complexes [47]. As the density of bacteria increases, AHL gradually accumulates. As the population density attains a critical “quorum”, these AHL molecules surpass a threshold concentration, thereby banding with receptors known as R proteins, subsequently governing the transcription of target genes through their intricate interaction with multiple promoters or their detachment from their corresponding promoter regions [48]. A jump-start mechanism exists for quorum sensing, and when the signal is inhibited, for example by group interference, the pathway can still be activated [49].

Some acidophiles, such as *A. ferrooxidans*, possess a fully functional AfeI/R-type QS system, capable of synthesizing and responding to AIs. Two QS systems have been found in *A. ferrooxidans*. The first set (AHL-QS) is typical of the LuxI/R type, including the functional genes *afeI*, *afeR*, and the unknown functional gene *orf3*: *afeI* encodes an AHL synthase that synthesizes AHL. The gene *afeR* encodes an AHL receptor protein, which is the transcriptional regulator of the LuxR family of proteins [43]. It has been demonstrated that *afeR* exhibits a specific binding affinity towards the regulatory region of the *afeI* gene [50]. Moreover, *afeI* and *afeR* are preferentially expressed early in the stabilization phase [43]. The cis-regulatory components of *afeR* and *orf3* coincide with the -35 promoter region, suggesting that AfeR may be capable of self-regulation [51]. The second set is an atypical QS system containing *glyQ*, *glyS*, *gph*, and *act* genes; the four genes are located in the same operon and encode aminoacetic acid tRNA synthetase, phosphorylase, and acyltransferase [39].

Other acidophiles also contain the AfeI homologous protein with the ability to synthesize AIs. The presence of an AHL-QS locus in *L. ferrooxidans* differs from the structural organization of *A. ferrooxidans* [45]. This locus consists of two convergent genes for the leptospiral *lttR* and *lttI*, and a third ORF has been found in the *lttIR* intergenic region [45]. A SdiA-like protein has been identified in *Acidithiobacillus thiooxidans* as strongly likely to take part in QS transcriptional regulation [44]. SdiA is an orphan QS transcriptional regulator, independent of the AHL synthase. Orphan QS transcriptional regulators can perceive AHLs from other bacteria in microbial consortia and are key molecules for QS flexibility and adaptation [52].

**Figure 1:**
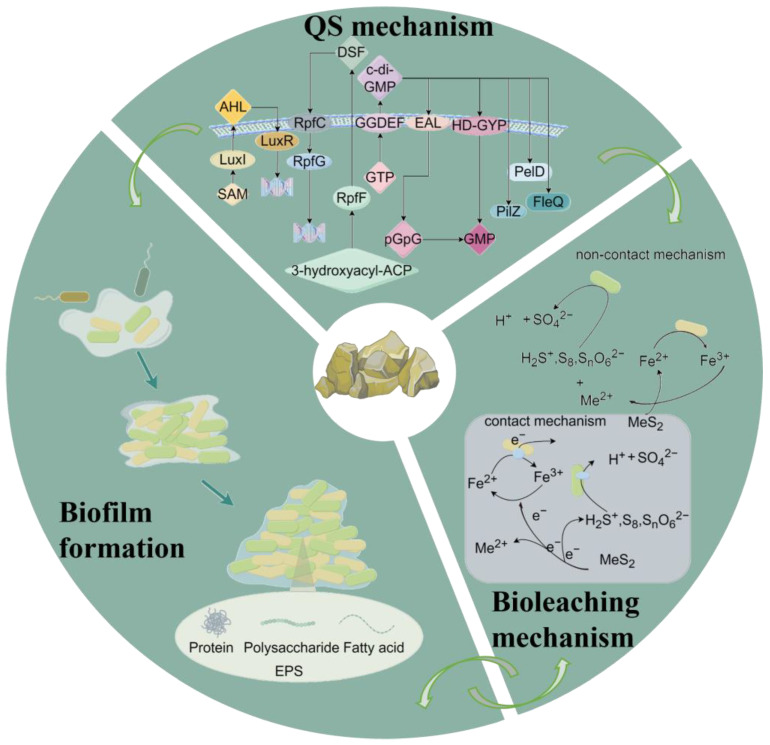
Regulation mechanism of bioleaching based on quorum sensing. QS has been extensively studied and proven to affect various aspects of cell morphology, community structure, biofilm formation, and cell metabolism in regulating the bioleaching process. Acronyms: SAM = S-adenosyl-L-methionine, EPS = extracellular polymers.

An acidophilic bacterium can produce multiple variants of AHL. The transport of the same AHL into the extracellular environment can cause the recognition of a variety of bacteria. *A. ferrooxidans* and *A. thioooxidans* produce AHL [45]. S-adenosyl-L-methionine is acylated by AHL synthase and reacts with an acyl–acyl carrier protein (acyl-ACP) to produce AHL [53] (Figure 1). Under different culture conditions, a single bacterium can produce 24 different kinds of AHLs [54]. *A. ferrooxidans* synthesizes AHLs with acyl chains ranging from 8 to 16 carbon atoms in length, which exhibit oxygen and hydroxyl substitutions at the third carbon position [55]. The preference for unsubstituted, 3-oxo, or 3-hydroxy acyl-ACP substrates is believed to stem from the inherent selectivity of the AHL synthase towards specific subgroups of acyl-ACP compounds generated through the reductive condensation cycle of fatty acid biosynthesis [56]. *A. thioooxidans* produces C_10_- and C_12_-HSL [17]. C_12_- and C_14_-HSL synthesized by *A. ferrooxidans* show detectable increases in the early stabilization period [43].

### 2.2. Diffusible Signal Factor System

DSF-mediated intraspecific communication pathways belong to different taxa [57]. In *Xcc*, DSF ((Z)-11-methyl-2-dodecenoic Acid) synthesis relies on adjacent *rpfF* and *rpfB* genes in the *rpf* gene cluster, which encode the putative enoyl-CoA hydrase and the putative long-chain fatty acid acyl-CoA ligase [58]. Bacteria produce DSF through the bifunctional enzyme RpfF and the classical fatty acid synthesis pathway, and RpfC controls synthesis through its receiver domain to form a complex with RpfF [59]. After a QS response, the system can be induced to restore DSF levels to pre-QS levels [60].

Genes encoding DSF family signaling-specific two-component systems or response regulators and genes suitable for DSF signaling sensing have been discerned within the genomes of *Acidithiobacillus caldus, L. ferriphilum*, *L. ferrooxidans,* and *Sulfobacillus thermosulfidooxidans* [40,61]. The *L. ferriphilum* DSM 14647^T^ genome contains a gene cluster containing the DSF system, which consists of the gene *rpfF* encoding a diffusion signaling factor synthase, genes encoding *rpfC* homologs, and their respective two-component systems of response modifier genes *rpfG* [62]. The biosynthesis of the diffusible signal factor in *Leptospirillum spp.* represents a sophisticated ecological niche defense mechanism aimed at safeguarding its habitat and countering antagonistic microorganisms.

### 2.3. Cyclic Dimeric Guanosine Monophosphate System

The c-di-GMP pathway proves to be the most versatile second messenger in acidophiles [63]. The regulation of c-di-GMP concentration relies on the antagonistic actions of di-guanosine cyclases (DGCs) and phosphodiesterases (PDEs) [63]. This pathway converts both environmental conditions and intracellular signals into different concentrations of c-di-GMP, which regulates flagellar motility, substrate attachment, and biofilm development by binding to different effector proteins, such as the PilZ domain, FleQ, and PelD proteins [63].

*A. ferrooxidans*, *A. thiooxidans*, and *A. caldus* have functional c-di-GMP pathways [46,64]. Several putative ORFs encoding DGC, PDE, and effector proteins have been identified in the study of *Acidithiobacillus* species. Cyclic-di-GMP is synthesized by DGCs, whereas PDEs catalyze the degradation of c-di-GMP to 5′-phosphoguanylyl-(3′-5′)-guanosine (pGpG) and GMP [65,66]. DGC activity resides in the GGDEF domain, while PDE activity resides in two unrelated domains: EAL and HD-GYP [63]. Two c-di-GMP effector proteins with PilZ domains have been identified in the genome of *A. ferrooxidans*, which showcases their pivotal role in controlling biofilm formation and mineral colonization [41]. The *bcs* operon encoding cellulose synthesis is widely found in acidophiles, while the PEL operon encoding PEL extracellular polysaccharide synthesis is only found in *A. caldus* and *A. thiooxidans* [44]. They are involved in biofilm formation in the *Acidithiobacillus* species [41,67].

## 3. Interactions between Different Systems

The AHL-QS system and the DSF system interact with each other in acidophiles. The AHL-QS-related genes and the DSF system are present in *L. ferriphilum*, where genes encoding the DSF system are expressed at high levels, whereas RNA transcripts of the genes encoding orphan LuxR proteins are very low [62]. The number of *rpfF* transcripts increases after AHL addition to *L. ferriphilum* [40]. The level of *flrB* (influencing bacterial adhesion, motility, biofilm formation, and EPS production) transcripts is reduced in *L. ferriphilum* after treatments involving DSF/BDSF and AHLs [40]. AHL-QS affects the expression of DSF synthesis genes and regulates the transcription of downstream genes such as *flrB*. Whether there is a specific cascade, cooperation, and competition relationship between the AHL system and the DSF system remains to be explored.

QS can either promote or inhibit biofilm growth, depending on the existence or nonexistence of environmental cues that influence c-di-GMP signaling [68]. The GGDEF/EAL domain is preferentially expressed in the biofilm state of *Leptospirillum* spp. living with *A. ferrooxidans* [41]. An unknown AHL receptor binding 3-oxo-C_8_-HSL (N-(3-Oxooctanoyl)-L-homoserine lactone) can directly promote *pel* manipulator expression and facilitate the biosynthesis of PEL exopolysaccharides of *A. thiooxidans* [44]. QS systems and c-di-GMP pathway connections, under the influence of environmental signals and cell density, transform *A. ferrooxidans* from a planktonic to biofilm lifestyle [41].

The DSF system is associated with c-di-GMP degradation and synthesis. RpfG contains typical receptor structural, and HD-GYP structural, domains, and RpfG controls the expression of a panel of proteins with GGDEF or EAL domains that may be involved in the synthesis or degradation of c-di-GMP [66,69]. It has been found that the DSF system can activate the HD-GYP of the RpfG domain and enzymatically regulate c-di-GMP metabolism, bacterial signaling, and response to environmental signals [70,71].

## 4. The Role of QS in Bioleaching

QS has been extensively studied and proven to affect various aspects of cell morphology, community structure, biofilm formation, and cell metabolism in regulating the bioleaching process.

### 4.1. Cell Morphology

Flagellar gene clusters are distributed in almost all *Acidithiobacillus* species except *A. ferrooxidan* and *Acidithiobacillus ferridurans*, where sulfur oxidizers containing the *Sox* system tend to form flagella [72,73]. Certain acidophiles that have been isolated have flagella to enhance attachment to minerals [74,75]. A series of genes related to flagellar synthesis, such as *flgA~K* and *fliC*, have been identified in the downstream genes of the QS system of the strain, which can regulate bacterial motility and chemotaxis [76]. QS exerts an indisputable influence on the intricate process of flagella formation, subsequently impacting cellular locomotion and chemotaxis.

### 4.2. Community Structure

Secretion and response systems for AIs are universally distributed in bacterial communities [77]. Microorganisms perceive AIs and subsequently alter their behavior, consequently exerting influences on the collaborative and antagonistic dynamics among microorganisms, thereby engendering modifications within the intricate web of microbial assemblages [78].

QS affects bacterial community composition through a combination of mechanisms [79]. QS can regulate the production of “public goods” that provide public compounds or functions such as extracellular hydrolases or siderophores [80,81,82]. The coordination of enzyme production through QS ensures an optimal distribution of resources and energy utilization within the microbial community, maximizing the efficiency of metal extraction. In addition, QS regulatory mechanisms can affect microbial communities through the production of antimicrobial compounds [83,84]. Changes in bacterial community composition driven by QS may be due to the chemical properties of AIs themselves, including their antimicrobial activity and iron-chelating properties [85,86]. Unsaturated fatty acids have been identified as compounds possessing antimicrobial properties that function by perturbing the cellular membranes of Gram-positive bacteria [87]. Simultaneously, DSF also falls within this classification of lipids. The recognition and reaction of *L. ferriphilum* bacteria to DSF encompasses not only the regulation of the QS signal but also the acid stress response to uptake the DSF molecule itself.

### 4.3. Biofilm Formation

Biofilms exist as a protective barrier for microorganisms during bioleaching. By coordinating the attachment and detachment of cells, QS enables microbial communities to collectively attach to mineral surfaces, forming biofilms that can protect microorganisms from extreme environmental conditions [88,89]. Zhang et al. proposed a model for the biofilm formation of bacterial strains in bioleaching [90,91,92]: Firstly, the cells reversibly attach to the mineral surface while producing adherent EPS compounds [93,94]. Secondly, the cells are firmly attached to the pyrite surface and colonize it; small microcolonies can also be interconnected by EPS or cellular appendages [90,95]. Finally, large colonies and erosion pits form as the cell surface erodes. The cells are encapsulated in an EPS layer dominated by carbohydrates and proteins [90,96]. The different stages of biofilm formation in acidophiles are regulated by the QS system (Figure 1).

The regulation of biofilm formation encompasses bacteria movement, EPS production, intracellular signaling, and intercellular communication [97]. The QS network accounts for at least 4.5% of the *A. ferrooxidans* ATCC 23270^T^ genome, of which 42.5% are associated with biofilm formation [50]. The identification of genes in the synthesis of EPS conveys that the QS pathway of *A. ferrooxidans* could potentially be linked to biofilm formation [98]. The c-di-GMP pathway is likewise important in biofilm formation. The high levels of c-di-GMP in adhesive cells, as compared to planktonic cells, suggest the involvement of the c-di-GMP pathway in biofilm formation in *A. ferrooxidans* [41]. A similar phenomenon has been observed in *A. thiooxidans* as well [64]. In addition, DGCs of *A. thiooxidans* couple the GGDEF structural domain to n-terminal signaling structural domains involved in signaling perception at the extracellular, membrane, and extraplasmic levels [99]. This strongly suggests that *A. caldus* regulates group movement and adhesion to sulfur surfaces through the c-di-GMP pathway [100]. *A*. *thiooxidans* and *A. caldus* have multiple copies of the *fleQ* gene, and 3-oxo-C_8_ -HSL may promote transcription of the gene encoding *fleQ*, thereby inducing biosynthesis of PEL exopolysaccharides [44]. PeL-like polysaccharides give active play in *A. thiooxidans* attachment to solid energy substrates [64]. The *L. ferriphilum* genome has identified genes encoding the PilZ domain that are functionally associated with the biosynthesis and export of cellulose and EPS, suggesting that c-di-GMP metabolism drives *L. ferriphilum* EPS production [40]. The expression of the effector proteins PelD and PilZ promotes the formation of acidophilic biofilms [67].

### 4.4. Microbial Metabolism

QS is intricately linked to the metabolic processes of bacteria, whereby the expression of genes induced by AHLs governs the synthesis and breakdown of metabolites. Ranava et al. postulated that QS is a protected function that operates independently from direct metabolic pathways [101]. On the other hand, DSF facilitates the reinforcement of the metabolic system, bolstering defense against infiltration, facilitating Na^+^ extrusion, aiding in iron absorption, and clearing reactive oxygen species [102]. The influence of AI-2 on metabolism is equally noteworthy, as it notably enhances fatty acid elongation, amino acid metabolism, and phosphorus relay signaling [103]. In bioleaching, QS acts as a harmonizing conductor, facilitating the synchronization of various metabolic processes vital to the successful extraction of metals from ores.

The sulfur and iron metabolism of acidophiles is closely related to their rapid adaptation to nutrient deprivation and extreme survival environments. *A. ferrooxidans* has two different AHL-based QS mechanisms, one based on Act and the other on the AfeI/R QS system. The expression of the gene *act* is more up-regulated in an iron-containing medium than in a sulfur-containing medium. At the same time, the reverse is true for the AfeI/R QS system, which is more up-regulated in a sulfur-containing medium than in an iron-containing medium, suggesting that these signals are related to the ability of *A. ferrooxidans* to colonize and utilize different sulfur- and iron-containing minerals [39]. Differences in energy substrates may be responsible for the changes in AHLs. *A. ferrooxidans* synthesizes acyl HSLs with C-3 hydrogen and hydroxyl substituents in a sulfur-rich medium, whereas only 3-OH-HSLs are found in an Fe^2+^ -rich medium [104]. 3-OH-C_14_-HSL(N-(3-Hydroxytetradecanoyl)-DL-homoserine lactone) works when *A. ferrooxidans* is cultivated with sulfur, but has no effect on growth when *A. ferrooxidans* is cultivated with Fe^2+^. The QS system regulates the microbial ability to utilize, assimilate, and sequester iron by modulating the secretion of specific EPS components, Fe^3+^-reductase, siderophores, and other relevant iron uptake factors [105]. The substrate-based regulatory model of acidophiles may be an important mechanism for them to gain a competitive advantage in extreme environments.

## 5. Regulation of QS in Bioleaching

QS modulation is a phenomenon influenced by the concentration of molecules. The current modulation techniques aim to augment the abundance of AIs within bioleaching through direct or indirect mechanisms. According to the different sources of AIs, the existing QS-based regulatory methods can be divided into endogenous regulation and exogenous regulation.

### 5.1. Endogenous Regulation

At present, endogenous QS-based regulation in bioleaching is mainly achieved by constructing engineered strains. AHL-QS-overexpressing strains show significant differences in biofilm formation. The cultivation of *A. ferrooxidans* in pyrite and the overexpression of AHL-QS led to a significant increase in EPS synthesis [106]. Overexpression of the AHL-QS or the *afeI*-only gene strains shows enhanced adhesion to cobaltite [107]. Overexpression of *afeI* stimulates the synthesis of EPS, further enhancing cellular adhesion to and bioerosion of sulfur [104]. The cell growth and sulfur metabolism of the overexpression of the *afeI* strain are significantly higher than the control strain during the logarithmic growth phase when in a sulfur-containing medium [104]. The gene *afel* overexpression promotes the sulfur metabolism of cells in the pre- and mid-culture period, and genes involved in periplasmic and cytoplasmic sulfation, including the *hdr* operon, *doxDA* operon, and *cyo* genes, are up-regulated [104]. The concentration of AHLs is key to determining whether *afeI* plays a regulatory role. In a sulfur-containing medium, when the concentration of AHLs reaches a threshold, the AfeI/R system is activated, accelerating cell growth. However, the regulation of *A. ferrooxidans* by AfeI/R can be considered a potent “inhibitor” of cell growth and cell density when cultured with an iron-containing medium. Overexpression of *afeI* inhibits ferrous oxidation and the cell growth of *A. ferrooxidans* [104]. Genes involved in ferrous oxidation and electron transport, including *rus* operon and *pet* operon, are significantly down-regulated in *afel* overexpression strains [104]. Transcriptomic analysis has revealed the up-regulation or down-regulation of gene expression by AHLs, but the regulatory mechanisms of QS in acidophiles still need further exploration.

### 5.2. Exogenous Regulation

Compared with endogenous regulation, exogenous regulation can directly increase the concentration of AIs in biological leaching, thereby more directly activating QS regulation. AHL is the most widely studied class of AIs in exogenous regulation. Exogenous AIs play an important role in regulating bioleaching (Table 3).

#### 5.2.1. N-Acyl Homoserine Lactones

The difference in community composition has a significant effect on the leaching rate. Exogenous AHL leads to changes in the structure of bacterial communities, not only in AHL-mediated QS bacteria but also in other microorganisms that use auto-inducible peptides for communication [110,111,112]. Exogenous C_14_-HSL increases the adhesion of native *A. ferrooxidans* cells to pyrite in a mixed bioleaching community [98]. Exogenous AHLs modulate the microbial community structure to increase the final ratio of functional strains and facilitate the hydrolysis of difficult-to-biodegrade organics [110,113,114].

Early adsorption of cells is the key to biofilm formation [115]. The addition of AHL affects cell attachment by different species of acidophiles [17]. QS increases the attachment of *A. ferrooxidans* to pyrite and sulfur by the addition of the AHL molecule [98]. The AHL mixture inhibits *A. ferrooxidans* ATCC 23270 attachment to pyrite with a small but significant trend [55]. Similar research indicates that an AHL mixture consisting of hydroxy-AHL and oxo-AHL inhibits both the rate and degree of bacterial adhesion to pyrite [45].

EPS mainly comprises proteins, polysaccharides, fatty acids, and metal ions, and multiple components promote mineral leaching [116,117,118,119,120]. EPS interacts with iron-containing minerals through the formation of P-O-Fe bonds, thereby inducing microbial adhesion and EPS accumulation [97]. AHL molecules with long acyl chains promote EPS formation, as well as increasing *A. ferrooxidans* attachment on sulfur and pyrite surfaces [98,104]. Adding C_8_-, 3-oxo-C_8_-, or C_10_-AHL enhances *A. thiooxidans* biofilm formation on sulfur substrates [17]. AHL selectively promotes the synthesis of amino acids and promotes extracellular protein content [121]. AHL facilitates the synthesis of acidophile EPS and augments its resilience in arduous environments.

The addition of AHL affects iron–sulfur metabolism and bioleaching. The addition of an AHL mixture inhibits the biofilm formation of *Acidithiobacillus ferrivorans* SS3, *Acidiferrobacter* sp. SPIII/3, and *L. ferrooxidans* DSM 2391, with a decrease in pyrite leaching [108]. The carbon felt electrodes in the biobattery are pre-colonized by *A. ferrooxidans* ATCC 23270 by the addition of C_14_-AHLs and with Fe^2+^ as an electron donor, and their current output, from −0.31 A m^−2^, increases to −0.56 A m^−2^, suggesting that C_14_-HSL has a significant impact on *A. ferrooxidans* ATCC 23270 [122]. Exogenous AHLs enhance the ability of *Acidithiobacillus* and *Pseudomonas* to extract metals from discarded printed circuit boards [123]. An in-depth understanding of the mechanism of AHL-QS regulation of iron–sulfur metabolism in acidophiles could be a useful tool for the development of bioleaching [124]. Acidophiles may utilize the QS system to establish a co-evolutionary process that regulates responses from energy substrates to cell growth and population density, which is an important pathway for chemotrophic autotrophs to adapt to growth environments and gain ecological competitive advantages.

#### 5.2.2. N-Acyl Homoserine Lactones Analogues

Different AHL analogues promote or inhibit the attachment of acidophiles and the formation of EPS. The AHL analogue 4-phenyl-3-yloxy-HSL derivative decreases attachment levels upon addition, while some AHL analogues, such as 3-sulfonylamide-C_8_-HSL, induce an increase in attachment levels [45]. Tetrazole accelerates the adsorption of *A. ferrooxidans* to sulfur flakes and up-regulated *afeI* and *zwf* (involved in the intracellular levels of α-D-glucose-6 phosphate) gene expression [109]. Tetrazole 9c triggers the QS system by inducing gene expression that allows AHL efflux [50]. Meanwhile, tetrazole 9c inhibits several genes involved in carbohydrate metabolism, directing carbon flow to the synthesis of maltodextrin to increase adhesion, accumulating α-D-glucose-6 phosphate and α-D-glucose-1-phosphate associated with EPS precursor biosynthesis to promote biofilm formation [50].

#### 5.2.3. Diffusible Signal Factor

Exogenous DSF affects the leaching efficiency of acidophiles in bacterial attachment and iron–sulfur oxidation. Mineral-attached *A. caldus*, *L. ferriphilum*, and *S. thermosulfidooxidans* on mineral particles are reduced by the addition of DSF [61]. The reduced attachment rate to chalcopyrite may contribute to the reduced oxidation of Fe^2+^ [40]. The addition of DSF inhibits the oxidation of the soluble substrate Fe^2+^ or the insoluble substrate chalcopyrite in *L. ferriphilum*, *S. thermosulfidooxidans,* and *A. ferrooxidans* [61].

DSF mediates interspecific interactions. *L. ferriphilum* is dominant in the community after DSF addition [61]. DSF inhibits Fe^2+^ oxidation in post-inoculated *S. thermosuldooxidans*, contributing to a preference for the oxidation of inorganic sulfur compounds by *S. thermosuldooxidans* when co-cultured with *L. ferriphilum* [40]. *L. ferrooxidans* DSM 2391 and *Acidiferrobacter* sp. SPIII/3 inhibit each other during leaching, and the inhibition between the strains can be partially overcome by the addition of a mixture of C_8_-, C_14_-, and C_1618_-HSL, which increases the pyrite leaching rate [108]. One of the reasons may be due to the high sensitivity of *Acidiferrobacter* sp. SPIII/3 to DSF/BDSF secreted by *L. ferrooxidans* to inhibit growth or even lead to cell lysis, whereas AHL may inhibit *L. ferrooxidans* [40]. The addition of DSF and AHL reduces the number of transcript counts of *L. ferriphilum* flagellar-related genes [40]. AHL and DSF synergistically regulate the adaptation of acidophiles to extreme environments and mineral leaching.

## 6. Research Needs and Future Direction

While previous studies have shown that QS-based bioleaching control methods are useful at laboratory scales, more needs to be done at the pilot and industrial scales. The existing endogenous and exogenous regulatory methods need to be optimized. In addition, it is necessary to understand the regulatory mechanism of QS in bioleaching for future technology development. While previous research has provided insights into the molecular mechanism and impact of QS on individual strains, there is a lack of literature on the intricate signaling communication among different species of bacterial communities. Additionally, a comprehensive investigation into strain-to-strain communication within the same QS system is warranted. Previous studies have primarily focused on the AHL and DSF systems as AIs systems, leaving room for further experimental evidence to confirm the existence of additional QS systems in acidophiles. Moreover, there is great value in exploring the communication and interconnectedness between different QS systems. Acidophiles still require a thorough understanding of their secretion of AIs and the interaction of genes related to iron–sulfur oxidation. To better understand the above mechanisms, it is necessary to synthesize microbial communities and study the response behavior of QS in mixed microbial systems. Finally, a model of the relationship between different types of QS systems and passivation film formation and biofilm development in bioleaching should be established, so that the whole bioleaching system affected by QS can be directly evaluated with specific indicators. The study of the QS system of acidophiles has helped to unravel the underlying mechanisms that control biofilm formation in bioleaching. Additionally, delving deeper into the underlying mechanisms of QS in bioleaching could potentially lead to the discovery of novel microbial species with enhanced metal solubilization capabilities. Furthermore, comprehending the QS system in acidophilics also advances our knowledge of other functional microorganisms.

## 7. Conclusions

In previous studies, the mechanism of the QS system in acidophiles and its role in biological leaching has been explored by constructing overexpression strains and adding exogenous AIs. However, the research is limited to the laboratory scale, and future research needs to develop to medium and large scales, such as column leaching and heap leaching. Moreover, the strains and QS systems studied are relatively limited; it is necessary to expand the scope of research, construct a coupling model of the QS system and bioleaching system in the microbial community, and explore a reproducible, easy-to-operate and low-cost method to improve bioleaching efficiency.

## Figures and Tables

**Table 1:** Iron–sulfur oxidizing microorganisms in bioleaching.

(a) iron metabolism				
Microorganisms	Regulatory genes	Enzyme	Electron transfer chain	References
*Acidithiobacillus ferrooxidans* ATCC 23270	*rusA/B*	Rusticyanin oxidase	1. Fe^2+^ → Rusticyanin oxidase → Cyc1 → *aa_3_* oxidase 2. Fe^2+^ → Rusticyanin oxidase → CycA1 → *bc_1_* complex 3. Fe^2+^→Cyt_579_ → Cytochrome c	[6,19,20,21,22]
	*iro*	Iron oxidase		
*A. ferrivorans* s DSM 22755	*fox cluster*	haem–copper terminal		
*Leptospirillum ferriphilum* DSM 17947		Cyc1		
*Sulfobacillus acidophilus* DSM 10332		CycA1		
*Sulfolobus tokodaii* JCM10545 *Acidiplasma aeolicum* DSM 18409 *Acidianus brierleyi* DSM 1651 *Metallosphaera sedula* DSM 5348		Cyt_579_		
(b) sulfur metabolism				
Microorganisms	Regulatory genes	Enzyme	Reaction	References
*Acidithiobacillus caldus* ATCC 51756 *A. thiobacillus* A01 *A. ferrooxidans* ATCC 23270 *A. ferrivorans* DSM 22755 *Sulfobacillus acidophilus* DSM 10332 *Acidianus copahuensis* ALE1 *Metallosphaera sedula* DSM 535	*tetH*	Tetrathionate hydrolase	S_4_O_6_^2−^ → S_2_O_3_^2−^ + SO_4_^2−^ + S^0^	
	*tsd*	Thiosulfate dehydrogenase	S_2_O_3_^2−^ → S_4_O_6_^2−^	
	*sqr*	Sulfide quinone reductase	H_2_S → S^0^	[19,20,23,24,25]
	*doxDA*	Thiosulfate: quinone oxidoreductase	S_2_O_3_^2−^ → S_4_O_6_^2–^	
	*sor*	Sulfur oxygenase reductase	S^0^ → H_2_S + SO_3_^2−^ + S_2_O_3_^2–^	
	*tst*	Thiosulfate sulfurtransferase	S_2_O_3_^2−^ → SO_3_^2−^ + S^0^	
	*hdrABC*	Heterodisulfide reductase complex	glutathione oxidized → glutathione + SO_3_ ^2−^	
	*sat/cysC*	Sulfate adenylyltransferase/adenylylsulfate kinase	adenosine phosphosulfate → SO_4_^2−^	
	*sar*	sulfite: acceptor oxidoreductase	SO_3_ ^2−^ → SO_4_^2−^	
	*SoxXYZAB*	Sox system	S^2−^/S^0^/S_2_O_3_^2−^/SO_3_^2−^ → SO_4_ ^2−^	

**Table 2:** Structure and function of major AIs in acidophiles. Acronyms: AIs = autoinducers, DGCs = diguanylate cyclases, PDEs = phosphodiesterases, AHLs = N-acyl-homoserine lactones, DSFs = diffusible signal factor, c-di-GMP = cyclic dimeric guanosine monophosphate.

AIs	Microorganisms	Typical Structures	Regulatory Proteins	Functions	References
AHLs	*A. ferrooxidans* ATCC 23270 *A. thiooxidans* DSMZ 504 *L. ferrooxidans* DSM 2391 * A. ferrivorans SS3 *	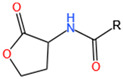	LuxI LuxR Act	1. Biofilm formation 2. Protein secretion 3. Flagellar movement	[17,38,39,43,44,45]
DSF	*L. ferrooxidans* DSM 2705 *L. ferriphilum* DSM 14647	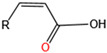	RpfG RpfF RpfC	1. Biofilm formation 2. Resistance	[40]
c-di-GMP	*A. ferrooxidans* ATCC 23270 *A. thiooxidans* ATCC 51756 *A. caldus* ATCC 19377	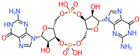	DGCs PDEs	1. Flagellar movement 2. Substrate adhesion 3. Biofilm formation	[46]

* indicates that the bacterium only contains orphan receptors.

**Table 3:** Quorum sensing-based exogenous regulation in acidophiles. Acronyms: HSL = homoserine lactone, OH-HSL = hydroxy-homoserine lactone, oxo-HSL = oxygen-homoserine lactone, 3-oxo-C8-HSL = N-(3-Oxooctanoyl)-L-homoserine lactone, BDSF = *Burkholderia* diffusible signal factor ((Z)-2-dodecenoic acid).

Exogenous Molecules	Acidophiles	Substrate	Results	References
OH-HSLs + oxo-HSLs	*A. ferrooxidans* ATCC 23270	pyrite	reduce adhesion	[55]
-HSL + OH-HSL	*A. ferrooxidans* ATCC 23270	sulfur	increase attachment	[98]
-HSL + OH-HSL + oxo-HSL/C_14_-HSL	*A. ferrooxidans* ATCC 23270	sulfur/pyrite	promote biofilm formation	[98]
-HSL + oxo -HSLs + OH -HSL	*A. ferrivorans* SS3	pyrite	1. inhibit biofilm formation 2. reduce the leaching rate	[108]
-HSL + oxo-HSL + OH-HSL	*L. ferrooxidans* DSM 2391	pyrite	1. inhibit biofilm formation 2. reduce the leaching rate	[108]
C_8_-HSL/3-oxo-C_8_-HSL/C_10_-HSL	*A. thiooxidans* DSM 14887	sulfur	promote biofilm formation	[17]
3-sulfonylamide-C_8_-HSL	*A. ferrooxidans* ATCC 23270	pyrite	increase attachment	[55]
4-phenyl-3-oxo-HSL	*A. ferrooxidans* ATCC 23270	pyrite	reduce adhesion	[55]
tetrazole	*A. ferrooxidans* ATCC 23270	sulfur	1. increase attachment 2. up-regulate *afeI* and *zwf* gene expression	[109]
tetrazole 9c	*A. ferrooxidans* ATCC 23270	sulfur	increase attachment	[50]
DSF + BDSF	*L. ferriphilum* DSM 14647	Fe^2+^/pyrite	1. inhibit iron oxidation 2. reduce adhesion	[40,61]
DSF + BDSF	*S. thermosulfidooxidans* DSM 9293	Fe^2+^/pyrite	1. inhibit iron oxidation 2. reduce adhesion	[40,61]
DSF + BDSF	*A. ferrooxidans* ATCC 23270	Fe^2+^	inhibit iron oxidation	[40,61]
DSF + BDSF	*A. caldus* DSM 8584	pyrite	reduce adhesion	[61]
DSF/BDSF + AHL	*L. ferriphilum* DSM 14647	Fe^2+^	reduce *flrB* transcription levels	[40]

## Data Availability

No new data were created or analyzed in this study. Data sharing is not applicable to this article.

## Abbreviations

**Abbreviation**	**Full Name**
QS	quorum sensing
EPS	extracellular polymers
AIs	autoinducers
AHL	N-acyl homoserine lactones
c-di-GMP	cyclic di-guanosine
DGCs	diguanylate cyclases
PDEs	phosphodiesterases
pGpG	5′-phosphoguanylyl-(3′-5′)-guanosine
DSF	diffusible signal factor
BDSF	*Burkholderia* diffusible signal factor
AHL-QS	AfeI/R-type QS system
SAM	S-adenosyl-L-methionine
acyl-ACP	acyl–acyl carrier protein
HSL	homoserine lactone
OH-HSL	hydroxy-homoserine lactone
oxo-HSL	oxygen-homoserine lactone
3-oxo-C_8_-HSL	N-(3-Oxooctanoyl)-L-homoserine lactone
3-OH-C_14_-HSL	N-(3-Hydroxytetradecanoyl)-DL-homoserine lactone
